# Involvement of leptin signaling in the development of cannabinoid CB2 receptor-dependent mirror image pain

**DOI:** 10.1038/s41598-018-28507-6

**Published:** 2018-07-17

**Authors:** Chihiro Nozaki, Elisa Nent, Andras Bilkei-Gorzo, Andreas Zimmer

**Affiliations:** Institute of Molecular Psychiatry, Medical Faculty, University of Bonn, 53127 Bonn, Germany

## Abstract

Neuropathic pain typically appears in a region innervated by an injured or diseased nerve and, in some instances, also on the contralateral side. This so-called mirror image pain is often observed in mice lacking CB2 receptors after sciatic nerve injury, but the underlying mechanisms for this phenotype largely remain unclear. Here we focused on peripheral leptin signaling, which modulates neuropathic pain development and interacts with the endocannabinoid system. Leptin production is induced at the site of nerve injury in CB2-deficient mice (CB2-KO) mice and wild type controls (WT). However, induction of leptin receptor expression was only observed in the injured nerve of CB2-KO mice. This was paralleled by a stimulation of the leptin receptor-downstream STAT3 signaling and an infiltration of F4/80-positive macrophages. Interestingly, an upregulation of leptin receptor expression STAT3 activity and macrophage infiltration was also observed on the non-injured nerve of CB2-KO mice thus reflecting the mirror image pain in CB2-KO animals. Importantly, perineurally-administered leptin-neutralizing antibodies reduced mechanical hyperalgesia, blocked mirror image pain and inhibited the recruitment of F4/80-positive macrophages. These results identify peripheral leptin signaling as an important modulator of CB2 signaling in neuropathic pain.

## Introduction

Neuropathic pain is elicited by an injury or inflammation of the nervous system. It typically appears in a region that is innervated by the affected nerve, but it can also develop on the contralateral side. Most experimental animal models of neuropathic pain do not show symptoms of contralateral hyperalgesia, although experimental protocols and genetic mouse lines in which mirror image pain can be induced have been described^1,2^, such as mice with a genetic deletion of the cannabinoid CB2 receptor^3^. This receptor is mostly expressed on immune cells^4^, whereas neurons prominently express cannabinoid CB1 receptors, although CB2 is also present on some neurons at very low levels^5,6^. Both are activated by the endocannabinoids 2-arachidonoylglycerol (2-AG) and arachidonoylethanolamide (AEA), as well as the phytocannabinoid Δ9-tetrahydocannabinol produced by *Cannabis sativa* plants^7^. The CB2 phenotype was due to the deletion of CB2 receptors from bone marrow-derived immune cells^8^ and dependent on an enhanced interferon-γ response. Double knockout mice lacking CB2 receptors and interferon-γ showed no contralateral hyperalgesia^8^.

To further elucidate the mechanism by which CB2-mediated signaling mediates neuropathic pain responses and mirror image pain, we focused our attention now on leptin, an adipocytokine that is best known for its role as a regulator of energy balance. Leptin is also involved in neurological pathologies and interacts with the endocannabinoid system^9^. Thus, nerve injury stimulates leptin release from adipocytes in peripheral nerves and activates infiltrated macrophages via leptin receptors, which leads to the increase production of iNOS, COX-2 and MMP-9^10^. Pharmacological inhibition of leptin signaling in the spinal cord^11^ or peripheral nerves^10^ attenuated neuropathic pain. It should be noted that hyperalgesia after acute nerve injury, which is promoted by leptin, is not detrimental as such, but rather aids in the recuperation process. In animal models of traumatic brain injury or stroke, leptin was also neuroprotective, improving neurological deficits and axonal injury markers^12^. Altogether these findings indicate that leptin production after neuronal injury enhances the healing process.

Several lines of evidence indicate that the beneficial effects of leptin involve CB2-dependent endocannabinoid signaling. Thus, leptin enhanced the expression of CB2 receptors in a stroke model^13^ and, vice versa, CB2 agonists stimulated expression of leptin in a paclitaxel-induced neuropathy model^14^. Blockade of CB2 receptors inhibited the neuroprotective effects of leptin^9,15^. Interactions between CB2 and leptin signaling were also described in the kidney^16^ and adipose tissue^17^.

We therefore investigated here the potential modulation of CB2-dependent mirror image pain by leptin signaling. We show that leptin receptor expression and downstream signaling pathways are enhanced in CB2 knockout mice after peripheral nerve injury and demonstrate that the peripheral blockade of leptin signaling with leptin-neutralizing antibodies completely blocked the development of contralateral hyperalgesia.

## Results

### Nerve injury induced robust leptin receptor expression in CB2-KO animals

**Figure 1:**
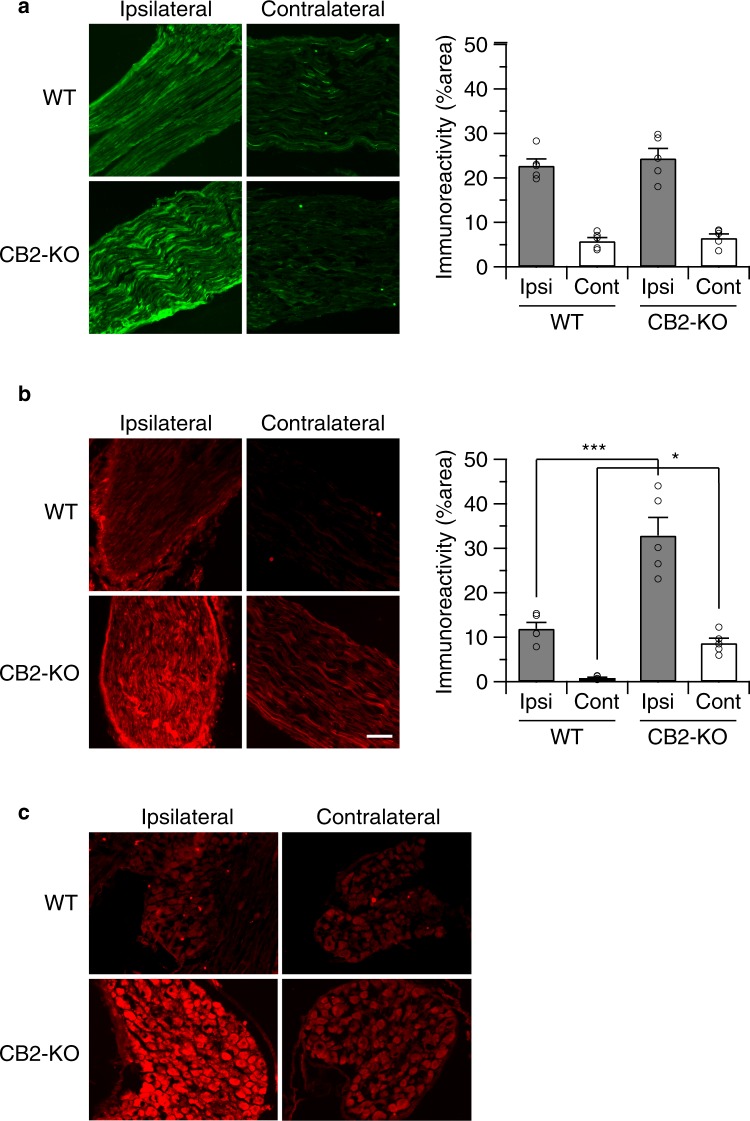
Leptin receptor expression was upregulated in nerve-injured CB2-KO animals 14 days after the surgery. Immunostaining of sciatic nerves using leptin (**a**) and leptin receptor (**b**) specific antibodies. Shown are the areas just next to the injured site (ipsilateral) or middle of dissected nerve (contralateral). Bars indicate a length of 100 μm. Leptin receptor expression was robustly increased in injured nerve of CB2-KO mice and a slight upregulation of leptin receptor expression was also observed on the contralateral nerve in CB2-KOs, but not in WT animals. The immunoreactivity was quantified (1 section/animal, n = 5) and expressed as percentage of positive signals to the stained area (mean ± SEM). A significant genotype effect is indicated by *p < 0.05 or ***p < 0.001, compared with respective WT controls (two-way ANOVA followed by Sidak’s multiple comparisons test). (**c**) A prominent induction of Leptin receptor expression was also observed in dorsal root ganglia of CB2-KO mice ipsilateral to the nerve injury.

To investigate the contribution of leptin activity on partial nerve ligation (PNL)-induced neuropathic pain, we first examined whether leptin or leptin receptor expression was modified by nerve injury. As shown in Fig. 1a, there was a strong leptin signal at the injured sciatic nerve 14 days after the ligation, whereas the uninjured nerve on the contralateral site showed only a weak signal. This indicates that leptin expression was induced by the nerve injury. The leptin signal was similar in WT and CB2-KO mice (p = 0.4334, also see Table 1). However, robust leptin receptor signal upregulation was observed in the both ipsilateral injured and contralateral non-injured nerve of CB2-KO mice, compared to those of WT animals (Fig. 1b, ipsilateral: p < 0.0001, contralateral: p = 0.0459, WT vs. CB2-KO). A similar pattern of leptin receptor induction was also observed in dorsal root ganglia.

**Table 1:** Power analysis.

Figure	Power achieved			Effect size (f)			Effect size for p < 0.05
	side	genotype	interaction	side	genotype	interaction	
Fig. 1a	0.9999	0.0605	0.0514	3.013	0.202	0.076	β < 0.2; n = 5: 1.358
Fig. 1b	0.9940	0.9396	0.2591	1.991	1.627	0.742	β < 0.2; n = 5: 1.358
Fig. 2	1.0000*	0.9839	0.9999	3.432*	1.076	1.447	β < 0.2; n = 5: 0.824
Fig. 3	0.7656	0.9998	0.0756	1.309	2.402	0.307	β < 0.2; n = 5: 1.358
Fig. 4a	1.0000	0.9984	0.0839	4.368	2.154	0.349	β < 0.2; n = 5: 1.358
Fig. 4b	0.9999	0.9972	0.864	2.684	2.088	1.4549	β < 0.2; n = 5: 1.358
Fig. 4c	0.9999	0.9976	0.9821	2.540	2.107	1.835	β < 0.2; n = 5: 1.358
	time	genotype/group	interaction	time	genotype	interaction	
Fig. 5a	1.0000	0.9999	1.0000	2.058	2.058	0.9454	β < 0.2; n = 29: 0.520
Fig. 5b	1.0000	0.9999	1.0000	0.794	1.019	1.0186	β < 0.2; n = 29: 0.520
	side	genotype	interaction	side	genotype	interaction	
Fig. 6a	0.9956	0.9999	0.9961	2.031	2.832	2.049	β < 0.2; n = 5: 1.358
Fig. 6b	0.5400	0.1057	0.1381	1.061	0.433	0.5244	β < 0.2; n = 5: 1.358
Fig. 6c	0.4020	0.0667	0.0763	0.916	0.254	0.311	β < 0.2; n = 5: 1.358
Fig. 7a	0.9982	1.0000	0.8958	2.139	3.282	1.515	β < 0.2; n = 5: 1.358
Fig. 7b	0.9988	0.9991	0.9658	2.184	2.215	1.730	β < 0.2; n = 5: 1.358
Fig. 7c	0.9792	0.9429	0.8201	1.812	1.638	1.384	β < 0.2; n = 5: 1.358
	treatment	genotype	interaction	treatment	genotype	interaction	
Figs 6/7 ipsi	1.0000	0.8878	0.0611	4.321	0.751	0.108	β < 0.2; n = 5: 0.665
Figs 6/7 contra	1.0000	1.0000	1.0000	2.238	3.314	2.415	β < 0.2; n = 5: 0.665

^*^These factors are calculated for side x time.

**Figure 2:**
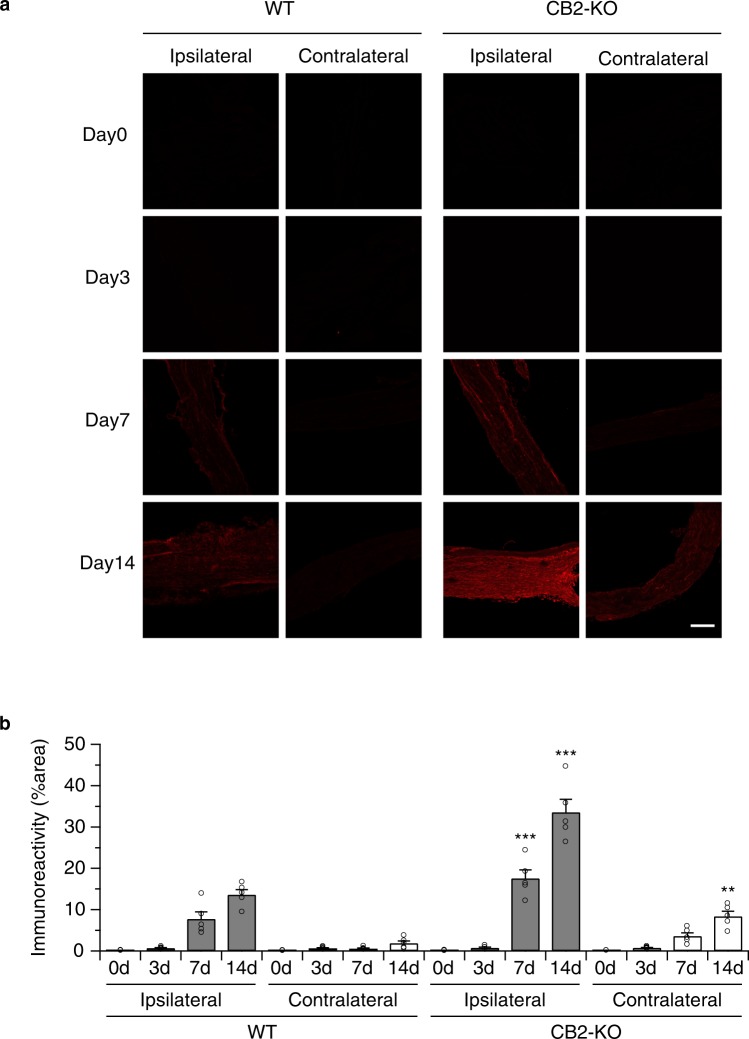
Leptin receptor upregulation on sciatic nerve depends on the days post-surgery. Left panels, WT mice; right panels, CB2 knockouts, 3, 7 and 14 days after the nerve injury stained with anti-leptin receptor antibody. Starting 7-days after the surgery, leptin receptor expression increased on the injured nerve of CB2 knockouts and was further increased in nerve-injured CB2 knockouts after 14 postoperative days. A slight upregulation of leptin receptors was also seen on the non-injured nerve of CB2 knockouts at this time point. Leptin receptor upregulation at 14 postoperative days was further observed in injured nerve of WT animals. Quantified immunoreactivity is expressed as percentage of positive signals to the stained area (mean ± SEM). A significant genotype effect is indicated by **p < 0.01 or ***p < 0.001, compared with respective 0-days controls (two-way ANOVA followed by Sidak’s multiple comparisons test), n = 5. Figure 3. Nerve injury-induced STAT3 phosphorylation in peripheral nerve was enhanced in CB2 deficient animals, as evaluated by STAT3 and pSTAT3 western blotting of nerve samples 14-days after the surgery. Quantified data were normalised to ß-actin and expressed as means ± SEM of 5 mice/group. Open column, WT animals; closed column, CB2 knockout animals. A significant surgery or genotype effect is indicated by *p < 0.05 or ***p < 0.001, compared with respective WT controls (two-way ANOVA followed by Sidak’s multiple comparisons test).

To determine at which time after the PNL leptin receptor expression was induced, we analyzed animals at days 3, 7 and 14 after PNL. We found leptin receptor expression only in animals with fully developed neuropathic pain (Fig. 2), but not in the early phase of nerve injury (3 and 7 days post-surgery). In this experiment we also found a slight upregulation of leptin receptor expression on the non-injured nerve of CB2-KO mice at day 14 after PNL (p = 0.0012).

### STAT3 activity was upregulated in sciatic nerves after PNL in CB2-KO animals

**Figure 3:**
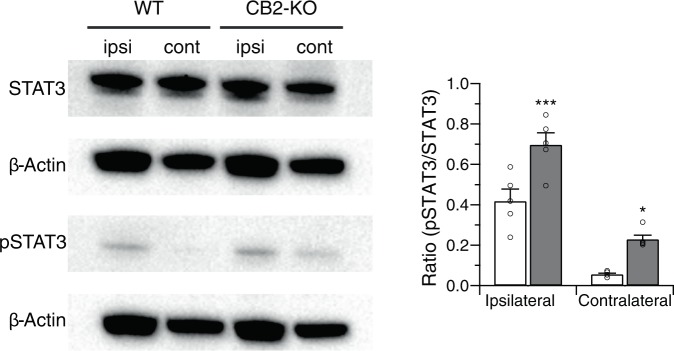
Nerve injury-induced STAT3 phosphorylation in peripheral nerve was enhanced in CB2 deficient animals, as evaluated by STAT3 and pSTAT3 western blotting of nerve samples 14-days after the surgery. Quantified data were normalised to ß-actin and expressed as means ± SEM of 5 mice/group. Open column, WT animals; closed column, CB2 knockout animals. A significant surgery or genotype effect is indicated by * p < 0.05 or *** p < 0.001, compared with respective WT controls (two-way ANOVA followed by Sidak’s multiple comparisons test).

Leptin receptor activation is typically followed by the phosphorylation of STAT3. We therefore evaluated STAT3 phosphorylation in sciatic nerve tissue after PNL. We observed an elevation in phosphorylated STAT3 on the ipsilateral side of the nerve injury in WT and CB2-KO animals (Fig. 3). However, the elevation of STAT3 phosphorylation was significantly higher in CB2-KO mice (p = 0.0006). Interestingly, we also found a slightly higher STAT3 phosphorylation in the non-injured contralateral nerve of CB2-KO mice (p = 0.0238). These results suggest that the upregulated leptin receptor levels in CB2-KO mice resulted in enhanced STAT3 signaling.

### Leptin receptors were induced on F4/80 positive macrophages

**Figure 4:**
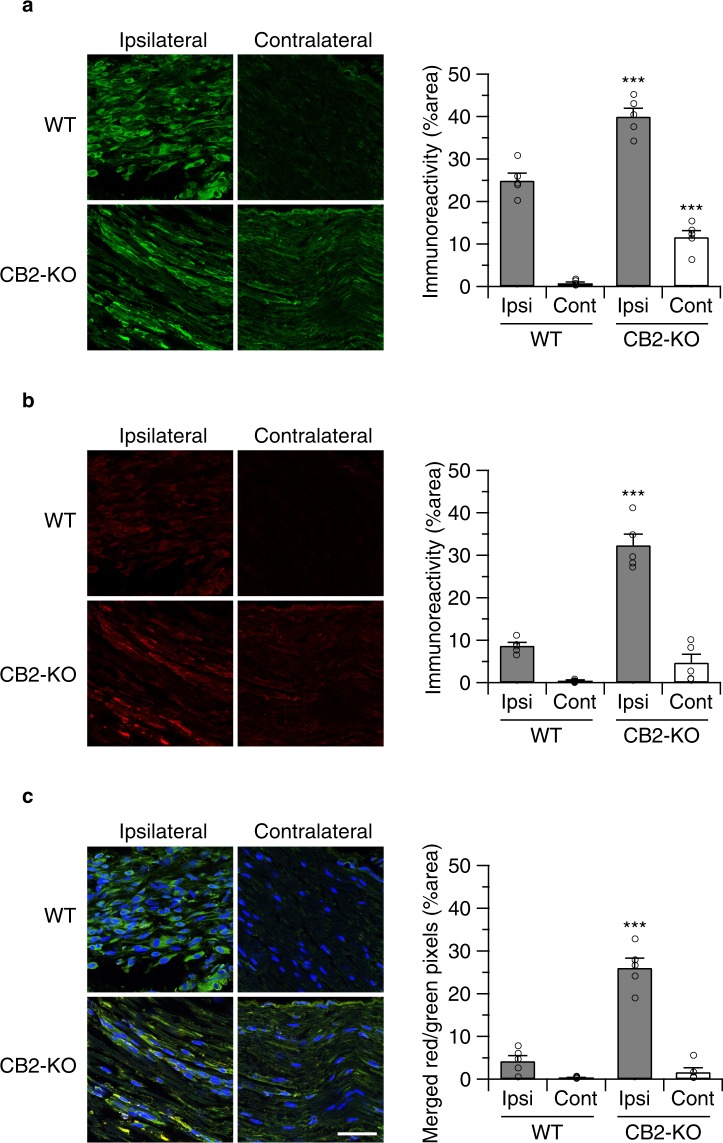
CB2-KO mice show enhanced nerve injury-induced macrophage recruitment in peripheral nerves. Shown are immunostaining nerve tissues with F4/80 (**a**) and leptin receptor (**b**) 14 days after the surgery. Bars indicate a length of 100 μm, except for bars on close-up image indicating a length of 50 μm. Merged image (**c**) show that leptin receptor signals overlap with F4/80 signals,indicating infiltrated macrophages were expressing leptin receptors in CB2-KO mice. Quantified immunoreactivity is expressed as percentage of positive signals to the stained area (mean ± SEM). A significant genotype effect is indicated by ***p < 0.001, compared with respective WT controls (two-way ANOVA followed by Sidak’s multiple comparisons test), n = 5.

We next evaluated macrophage infiltration after PNL, because these cells are known to express leptin receptors. We observed a stronger signal from F4/80 positive macrophages on the injured nerve (ipsilateral) compared to the non-injured nerve (contralateral) of CB2-KO and WT animals (Fig. 4). Again, the induction of leptin receptor signal as well as F4/80 signal was stronger in CB2-KO mice (leptin receptor: p < 0.0001, F4/80: p < 0.0001). Furthermore, an F4/80 signal was also observed in the non-injured nerve of CB2-KO mice, in contrast to WT animals (p = 0.0002). Most of F4/80 positive macrophages on the injured nerve in CB2-KO mice also expressed leptin receptors. Altogether, these results indicate that nerve injury-induced leptin signaling and macrophage infiltration was enhanced by the genetic deletion of CB2 receptors.

### Peripheral leptin activity modulated neuropathic pain development and macrophage infiltration

**Figure 5:**
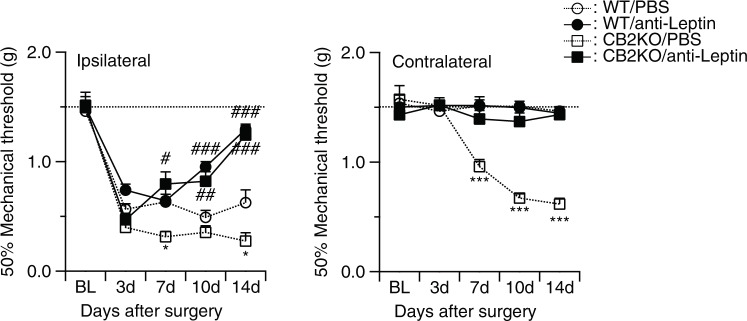
Analgesic effect of leptin-neutralizing antibody treatment (20 ng/mouse/day) in nerve-injured mice. Each measurement was conducted 24 hours after the last injection. Broken line indicates basal nociceptive thresholds before the surgery. Left panels, ipsilateral injured side; right panels, contralateral non-injured side. Leptin neutralizing antibodies significantly reduced the nerve injury-induced neuropathic pain in CB2-KO and WT mice. Data are expressed as means ± SEM of 7–8 mice/group. Significance is indicated by *p < 0.05, **p < 0.001, ***p < 0.0001 for genotype effect and #p < 0.05, #p < 0.001, #p < 0.0001 for the drug effect, three-way ANOVA followed by Tukey’s multiple comparisons test.

We next investigated whether peripheral leptin signaling contributed to the enhanced manifestation of neuropathic pain in CB2-KO mice. For this, we injected a neutralizing leptin antibody (20 ng/mouse/day^10^) perineurally at the ipsilateral side of the nerve injury and measured tactile allodynia using von-Frey filaments (Fig. 5). In vehicle treated mice, we found a pronounced allodynia in WT and CB2-KO mice already 3 days after PNL. Allodynia was significantly stronger in CB2-KO compared to WT mice (F(3,25) = 22.75, p < 0.0001). CB2-KO animals also developed a contralateral allodynia, starting at day 7 after PNL, as reported previously (F(3,26) = 11.81, p < 0.0001). The neutralizing anti-leptin antibody strongly reduced tactile allodynia in WT animals after 10 days of treatment (p = 0.0006 on day 10; p < 0.0001 on day 14, PBS-treated WT vs. drug-treated WT). In CB2-KO mice, we already observed a significant treatment effect on day 7 (p = 0.0017 on day 7; p < 0.0001 on day 10 and 14, PBS-treated CB2-KO vs. drug-treated CB2-KO). Importantly, there was no genotype difference after the anti-leptin treatment (p = 0.9663, drug-treated WT vs. drug-treated CB2-KO on day 14). Additionally, the ipsilateral injection of the anti-leptin antibody completely prevented the development of contralateral allodynia in CB2-KO animals (p = 0.7049, drug-treated WT vs. drug-treated CB2-KO on day 14).

**Figure 6:**
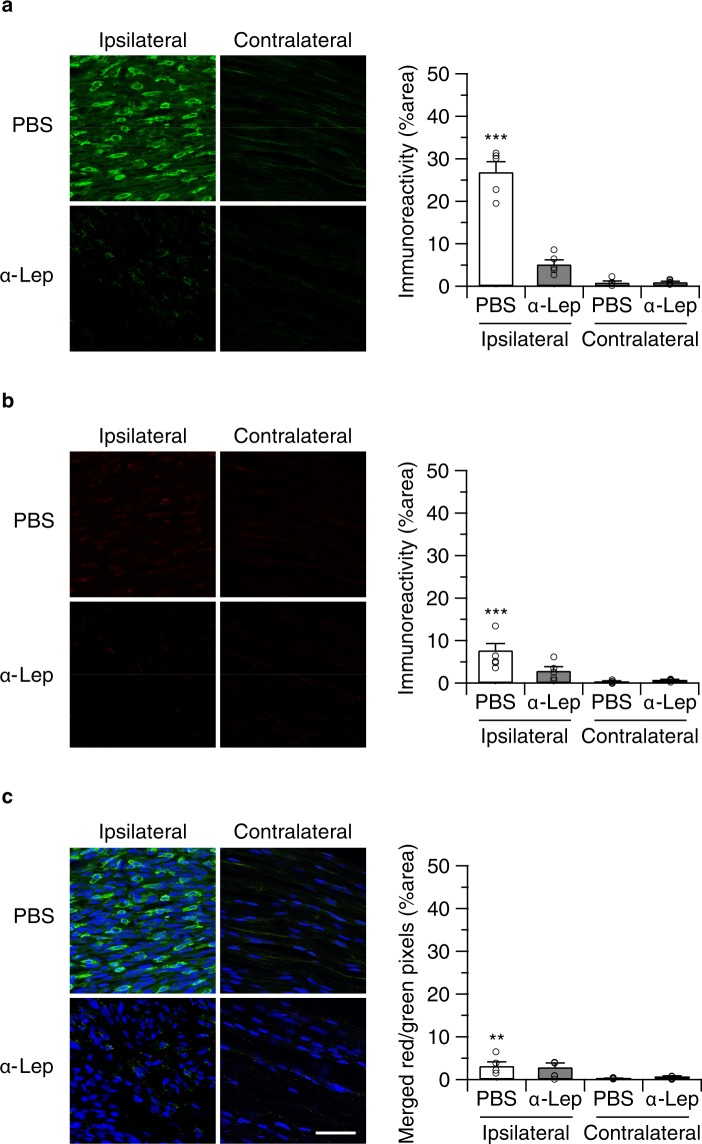
Perineurally administered leptin-neutralizing antibodies (20 ng/mouse/day) inhibited nerve injury-induced macrophage infiltration. Macrophage infiltration (**a**) as well as leptin receptor expression (**b**) was determined by immunohistochemistry 14 days after the surgery (24 hours after the final drug treatment). Bars indicate a length of 50 μm. (**c**) Merged images (green: F4/80, red: leptin receptor, blue: DAPI) showed that most of infiltrated macrophages in WT animals did not express leptin receptors. Quantified immunoreactivity is expressed as percentage of positive signals to the stained area (mean ± SEM). A significant treatment effect is indicated by **p < 0.01 or ***p < 0.001, compared with respective contralateral immunoreactivity (two-way ANOVA followed by Sidak’s multiple comparisons test), n = 5.

**Figure 7:**
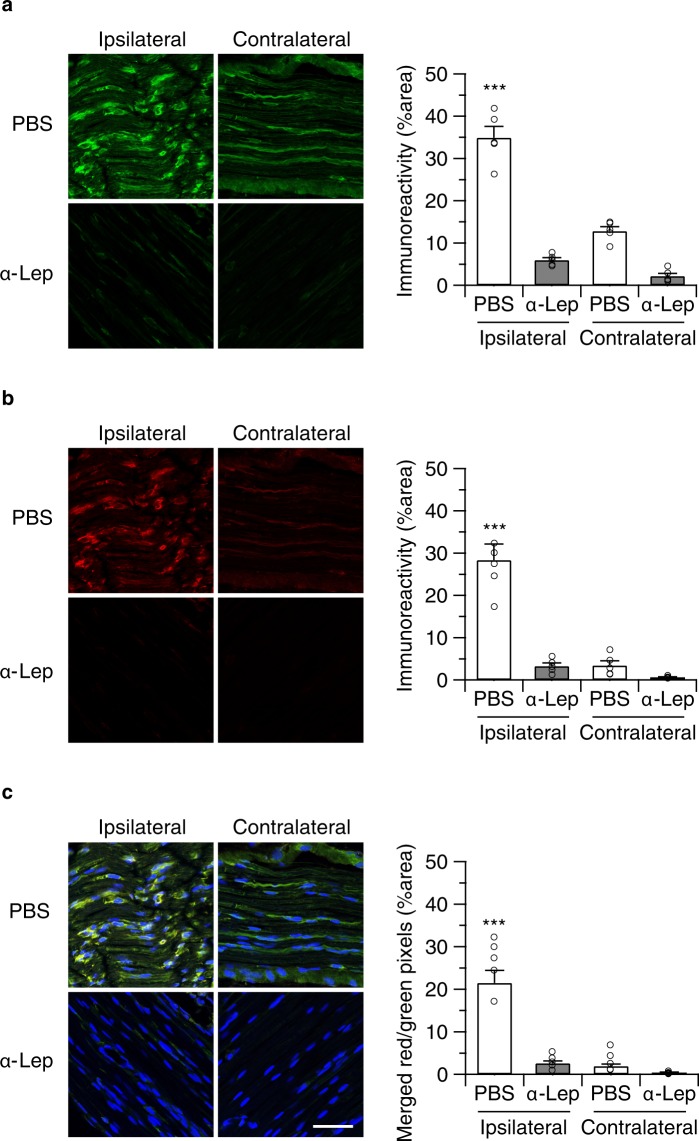
Perineurally administered leptin neutralizing antibodies (20 ng/mouse/day) inhibited nerve injury-induced macrophage infiltration in CB2-KO animals. F4/80 (**a**) and leptin receptor expression (**b**) as determined by immunohistochemistry 14 days after the surgery (24 hours after the final drug treatment). Bars indicate a length of 50 μm. (**c**) Merged images (green: F4/80, red: leptin receptor, blue: DAPI) showed that most of infiltrated macrophages in CB2-KO animals were leptin receptor positive. Quantified immunoreactivity is expressed as percentage of positive signals to the stained area (mean ± SEM). A significant treatment effect is indicated by *** p < 0.001, compared with respective contralateral immunoreactivity (two-way ANOVA followed by Sidak’s multiple comparisons test), n = 5.

We also determined if anti-leptin treatment modulated macrophage recruitment to the injured nerve in WT (Fig. 6) and CB2-KO animals (Fig. 7). In vehicle-injected controls, we found a similar pattern of F4/80 positive macrophages (ipsilateral: p = 0.0079, contralateral: p < 0.0001) and leptin receptor induction (ipsilateral: p < 0.0001, contralateral: p = 0.6673), as observed before. In contrast, there was a striking reduction of F4/80 positive macrophages in anti-leptin antibody-injected animals. Thus, the anti-leptin treatment completely abolished the genotype effects for macrophage recruitment (ipsilateral: p > 0.9999, contralateral: p = 0.9985) and leptin receptor expression (ipsilateral: p > 0.9999, contralateral: p > 0.9999).

## Discussion

In the present study, we evaluated leptin signaling as a potential CB2-downstream molecular mechanism contributing to the enhanced manifestation of neuropathic pain in CB2 deficient animals, which includes the development of contralateral hyperalgesia. We found that a partial ligation of the sciatic nerve resulted in an up-regulation of leptin peptide levels in the injured nerve. This was similar in WT and CB2-KO mice. CB2-KO mice also showed a strong induction of leptin receptor expression. The level of leptin receptor induction was much lower in WT mice and did not reach the level of significance. This genotype effect was reflected by a stronger activation of the leptin receptor-downstream signaling factor STAT3 in CB2-KOs. Our results are entirely consistent with the idea of a mutual positive interaction between cannabinoid and leptin signaling^9,13,15^. Thus, disrupting cannabinoid signaling via deletion of CB2 receptors is a likely cause for the observed deregulated leptin signaling. Strikingly, application of leptin-neutralizing antibodies at the site of nerve injury reduced tactile hyperalgesia in both genotypes and completely prevented the development of contralateral hyperalgesia in CB2-KO mice. Limitations of this study include the exclusion of female mice and the use of a constitutive CB2-KO model.

Mirror-image contralateral hyperalgesia is not commonly observed in most surgical neuropathic pain models. However, it has been described in a number of studies, for example in a rat chronic constriction injury model^1^ and after perisciatic zymosan injection^18^. The mechanisms leading to the development of contralateral hyperalgesia still remain to be fully elucidated, but central as well as peripheral contributing factors, such as endocannabinoid signaling, have been identified. Thus, sciatic nerve injury increased 2-AG levels in sciatic nerve tissue proximal to the lesion site^19^, in dorsal root ganglia^20^ and several brain regions involved in nociception^21^, as well as CB1 and CB2 receptor expression in the spinal cord^4,22^. Deletion of CB1 receptors did not affect the development of tactile hyperalgesia after sciatic nerve injury but aggravated the adverse effects on affective behaviors^23–25^. The deletion of CB2, in contrast, enhanced the manifestation of nociceptive hyperalgesia and produced a mirror image pain in several studies^3,8,26^. It is of note that the Lichtman laboratory did not observe a mirror image pain two months after a similar nerve injury in the same strain of mice^27^. It is possible that this difference was due to subtle experimental differences, but it could also indicate that the development of mirror image pain is only transient. While previous studies have emphasised the mirror image pain phenotype in CB2-KO mice, we demonstrate in this study that nerve-injured CB2-KO mice also display stronger hyperalgesia throughout neuropathic pain development.

Analyses of the underlying mechanisms pointed towards an enhanced spinal microglia activation in the absence of CB2 receptors, probably involving interferon gamma^8^. This is supported by our present observation of a robust infiltration of F4/80 positive macrophages at the site of nerve injury. Although macrophages are mainly recruited on the injured nerve in both WT and CB2-KO mice, a small number of F4/80 positive cells were also observed on the non-injured contralateral nerve in CB2-KO mice, but not in WT animals. Most upregulated leptin receptors observed in CB2 knockouts are localised on macrophages recruited to the site of nerve injury. It has previously been shown that leptin, which is likely produced by adipocytes in the epineurium of the injured sciatic nerve, stimulate receptor-expressing macrophages to produce algogenic mediators for the development of tactile allodynia^10^. This involves the stimulation of the JAK-STAT signaling pathway, which CB2-KO mice showed significant increase compare to WT controls. It is also of note that upregulation of leptin receptor and STAT3 phosphorylation was observed in WT mice only in the injured nerve, whereas in CB2 knockouts also showed such a response, albeit at much lower levels, on the non-injured contralateral nerve.

To examine if leptin signaling was essential for mirror-image pain development, we tested the effect of neutralizing leptin antibodies. We found that perineurally administered anti-leptin antibodies reduced tactile hyperalgesia after 10 days of treatment but had little or no effects on earlier treatment days. Furthermore, perineural anti-leptin antibodies also abolished leptin receptor upregulation and recruitment of F4/80 positive macrophages to the injured nerve. It thus seemed that leptin produced at the site of nerve injury is an essential mediator for the development of the nerve injury-induced neuroinflammatory response. Interestingly, although we only injected anti-leptin antibodies to the ipsilateral side, this treatment completely inhibited the mirror-image pain and contralateral neuroinflammation in CB2 knockouts. This result suggests that the contralateral mirror-image pain in the absence of CB2 signaling involved an enhanced peripheral leptin signaling and, consequently, an enhanced local inflammatory response. Enhanced neuroinflammation may cause the activation of spinal microglia^2^, which has been observed in spinal cord tissue of nerve-injured CB2-KO animals^3^, and the increased release of spinal proinflammatory cytokines, such as interferon gamma^8^. Thus, in summary, our findings strengthen the link between leptin and endocannabinoid signaling and demonstrate that this interaction is important for neuroinflammation and neuropathic pain development.

## Methods

### Animals

All experimental procedures and animal husbandry were carried out in accordance with the European Communities Council Directive of 22 September 2010 (directive 2010/63/UE), with the guidelines of the Committee for Research and Ethical issues of IASP published in PAIN, 1983; 16:109–110, and were approved by the local ethical committee (Landesamt für Natur, Umwelt und Verbraucherschutz in Nordrhein-Westfalen, Germany, AZ: 87–51.04.2011.A041–18). Particular efforts were made to minimize the number of mice and the pain they experienced. All mice were bred and maintained at the animal facility of the Medical Faculty at the University of Bonn, housed in a temperature (21 ± 1 °C) and humidity (55 ± 10%) controlled room with a 12-h light: 12-h dark cycle (light on between 08:00 h and 20:00 h). Food and water were available ad libitum except during behavioral observations. Different groups of male cannabinoid receptor 2 (CB2) knockout mice^28^ or male C57BL/6 J mice aging 7–9-weeks-old at the beginning of the experiments were used. CB2 knockout mice were backcrossed for more than 10 generations to C57BL/6 J mice and were therefore congenic for this genetic background. Animals were randomly assigned to experimental groups and blinded for genotype and treatment.

### Induction of surgical neuropathic pain

Partial nerve ligation (PNL)-induced neuropathic pain was produced by a tight ligation of approximately half the diameter of the left common sciatic nerve by 7–0 braid silk suture under deep inhalation anesthesia (2.5% isoflurane) according to the surgical method described previously^29^.

### Drugs

Topical inhibition of leptin signaling was conducted by perineural injection of leptin inhibitors. According to previous reports, leptin neutralizing antibody (R&D systems, Germany) has been used for perineural administration^10^. Leptin neutralizing antibody was dissolved in sterilized PBS, therefore same PBS has been used for vehicle injection as controls. Perineural injection (20 ng/mouse/day) has accompanied using the method described previously^10^, thus drugs were injected locally to perineural region of ipsilateral sciatic nerve. The first injection was done immediately after the surgery and was continued by single daily injections for 14-days in total. Every mouse was tested 24 h after the latest injection to avoid the acute pharmacological effect of inhibitors.

### Assessment of tactile sensitivity

The von-Frey filament up-down method^30^ was used to evaluate the tactile sensitivity following to the NTG injection. Briefly, mice were placed in an 8 cm × 10 cm × 15 cm Plexiglas box and the hind paw plantar surface was gently probed with a series of eight von Frey filaments with logarithmically incremental stiffness (0.008, 0.04, 0.07, 0.16, 0.40, 0.60, 1.00 and 2.00 g, purchased from Bioseb, Vitrolles, France). Stimuli were presented by probing at intervals of 5–10 seconds and sharp withdrawal or flinching of probed hind paw was considered as a positive response. The threshold of the response (50% tactile threshold) was calculated by using the up-down Excel program based on the equation formula described previously^30^, which was generously provided by Allan Basbaum’s laboratory (UCSF, San Francisco, CA, USA). Mice were habituated to their new experimental environment and handled for 3-days before starting the experiments. Baseline pain threshold was measured immediately before the surgery. Pain measurement was performed on 3, 7, 10 and 14 days after the surgery.

### Immunohistochemistry

Following to the behavioral experiment, 3 to 5 mice per group were deeply anesthetized with ketamine/xylazine (50/10 mg/kg) and then intracardially perfused with heparinized phosphate buffer (PB), followed by 4% formaldehyde. Both sciatic nerves (approximately 1 cm around the ligated area) were removed, fixed in 4% formaldehyde for 2 h, and cryopreserved in 30% sucrose solution at 4 °C. Nerve tissues were then cut to 10 μm thick longitudinal sections in a cryostat, mounted in Star frost-coated slides and kept under −80 °C until further immunostaining.

The slides were incubated in solutions containing anti-leptin antibody (1:100; R&D Systems), anti-leptin receptor antibody (1:40; R&D Systems) or anti-F4/80 antibody (1:100; Cedarlane) at 4 °C overnight, followed by incubation with Alexa488 or CY3 anti-goat secondary antibody (1:200; Jackson Immunoresearch, for anti-leptin and anti-leptin receptor) and Alexa488 anti-rat secondary antibody (1:200; Molecular Probes, for anti-F4/80) for 2 h at room temperature. After washing three times with PBS, the slides were mounted with Fluoromount-G containing DAPI. The sections were observed under the fluorescence microscope (Zeiss Axioplan) or a confocal microscope (LSM SP8, DMI 6000 CS, Leica). Images of two stained sections from both ipsilateral and contralateral nerve per each animal were acquired using a JVC (3-CCO) digital camera with NIS-Elements software (Nikon) for fluorescence imaging and LCS software (Leica) for confocal imaging. Tissue sections were examined with a 10 × objective (entire confocal overview), 20 × objective (fluorescence imaging) or a 63 × objective (detailed confocal close-up). Five images from individual slices were analyzed for each condition with ImageJ software (version 1.41, National Institutes of Health, USA) measuring the percentage of positive immunoreactivity to the stained area. Further image analysis was performed according to RGB (Red, Green and Blue) method described by Inman and colleagues^31^ using ImageJ macro ‘multiplecoloranalysis’, and the percentage of yellow pixels (Red-Green merge) to the stained area was obtained as “colocalized” positive signals.

### Western blotting

Western blotting of protein extracts from freshly dissected sciatic nerves was conducted with nerve tissue homogenized in 100 µl RIPA buffer (0.5 mM EDTA, 50 mM TrisHCl, 150 mM NaCl, 1% Nonidet P-40, 0.5% Sodium Deoxycholate, 0.1% SDS, pH 8.0) followed by 15 min centrifugation at 6500 rpm, 4 °C. Supernatant was collected and the protein concentration was evaluated by Pierce BCA Assay to prepare the samples with a final concentration of 20 µg/25 µl. Samples and molecular weight marker (Novex Sharp Prestained Protein Standard) were electrophoresed in NuPage Novex 4–12% Bis-Tris Protein Gel and transferred to the nitrocellulose membrane (Novex Gel Transfer Stacks) followed by 1-h blocking using 5% BSA in TBST with 0.1% Tween-20. The primary antibody for STAT3 (1:2000, Cell Signaling) or pSTAT3 (1:2000, Cell Signaling) was diluted in blocking buffer, applied and incubated overnight at 4 °C. After the washing with TBST, membrane was incubated with secondary antibody (Goat anti-rabbit HRP conjugated IgG, 1:5000, Jackson, USA) at room temperature for one hour followed by 2 min incubation ECL Western Blotting Substrate. Imaging of the membrane was performed with the BioRad ChemiDoc Imaging system using the ImageLab software (BioRad) for analysis of the picture. Loading control was obtained by b-actin blotting on stripped membrane.

### Data analysis

All data are presented as means ± SEM. Data analysis was performed using three-way (Fig. 5) or two-way ANOVA (all the others) followed by Sidak’s multiple comparisons test to determine statistically significant differences. A power analysis for all experiments was performed by G*Power 3.1.9.3 (University of Dusseldorf). The results are shown in Table 1.

## Electronic supplementary material

Supplementary Figure 1
