# Supplementary material for: Involvement of leptin signaling in the development of cannabinoid CB2 receptor-dependent mirror image pain
Source: Sci Rep. 2018 Jul 17;8:10827. doi: 10.1038/s41598-018-28507-6 (PMC6050271; doi:10.1038/s41598-018-28507-6)
Supplement: Supplementary file 1 — Supplementary Figure 1 [file 41598_2018_28507_MOESM1_ESM.pdf]

**Involvement of leptin signaling in the development of cannabinoid CB2 receptor-dependent mirror image pain**

Chihiro Nozaki, Elisa Nent, Andras Bilkei-Gorzo, Andreas Zimmer
Institute of Molecular Psychiatry, Medical Faculty, University of Bonn, 53127 Bonn, Germany

*Correspondence: Andreas Zimmer
Institute of Molecular Psychiatry, University of Bonn
Sigmund-Freud-Str. 25, 53127 Bonn, Germany
Phone: +49-228-688 5300
Fax: +49-228-688 5301
e-mail: neuro@uni-bonn.de

# Supplementary Information

Original images of western blots shown in Figure 3

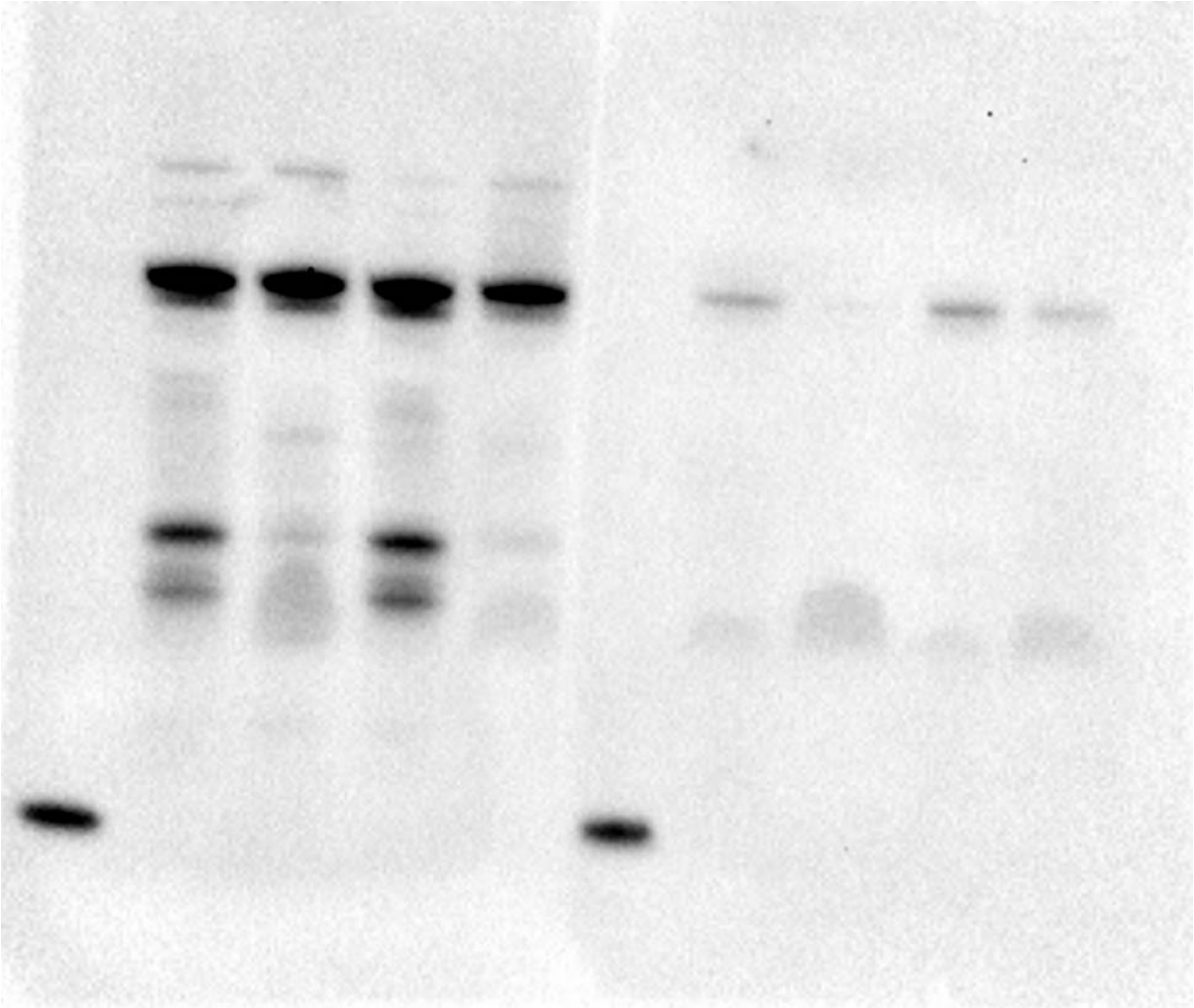

Stat3 / pStat3

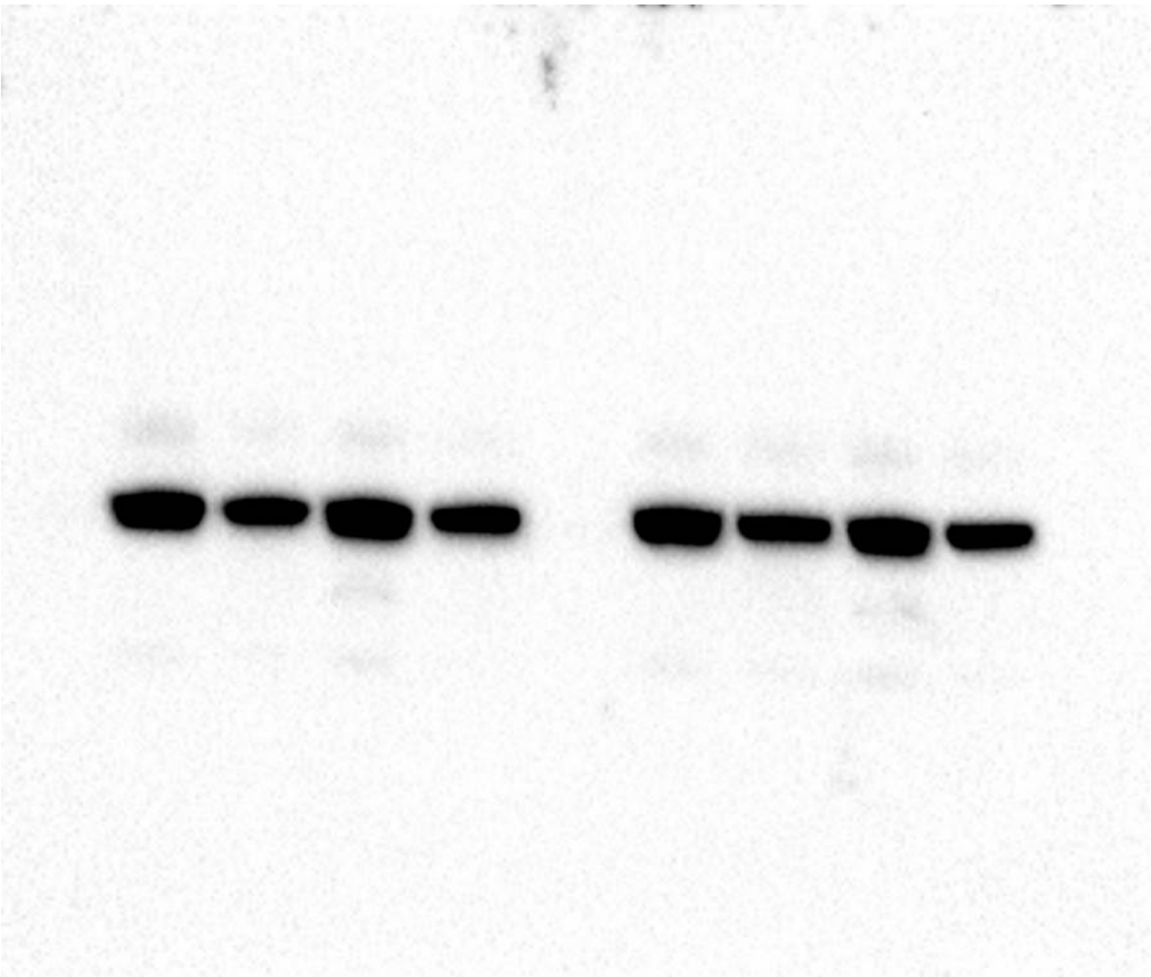



ß actin
